# Supplementary figures and images for: Acetate-producing bacterium Paenibacillus odorifer hampers lung cancer growth in lower respiratory tract: an in vitro study
Source: Microbiol Spectr. 2024 Oct 4;12(11):e00719-24. doi: 10.1128/spectrum.00719-24 (PMC11537125; doi:10.1128/spectrum.00719-24)

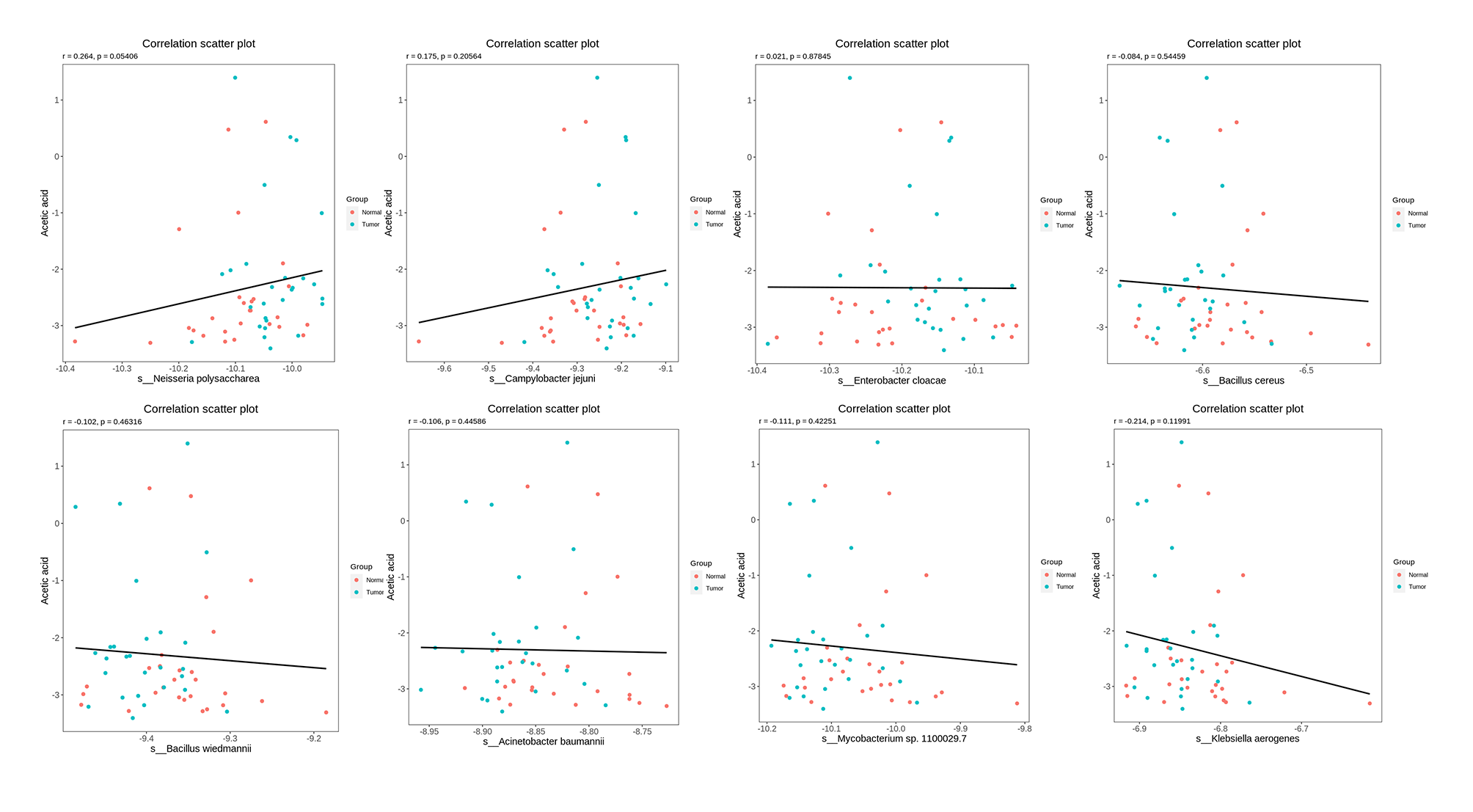

Supplement: Fig. S1 — Spearman correlation of candidate microbes and acetate acid. [file spectrum.00719-24-s0001.tif]
