# Supplementary material for: Acetate-producing bacterium Paenibacillus odorifer hampers lung cancer growth in lower respiratory tract: an in vitro study
Source: Microbiol Spectr. 2024 Oct 4;12(11):e00719-24. doi: 10.1128/spectrum.00719-24 (PMC11537125; doi:10.1128/spectrum.00719-24)
Supplement: Supplemental material — Supplementary figure legend. [file spectrum.00719-24-s0002.docx]

**Supplementary figure legend**

**Supplementary Figure 1** Spearman correlation of candidate microbes and acetate acid. R values and P values are listed on the top of each plots, respectively.
